# Age-related transcriptome changes in melanoma patients with tumor-positive sentinel lymph nodes

**DOI:** 10.18632/aging.202435

**Published:** 2020-12-29

**Authors:** Derek S. Menefee, Austin McMasters, Jianmin Pan, Xiaohong Li, Deyi Xiao, Sabine Waigel, Wolfgang Zacharias, Shesh N. Rai, Kelly M. McMasters, Hongying Hao

**Affiliations:** 1 The Hiram C. Polk, Jr., MD. Department of Surgery, University of Louisville School of Medicine, Louisville, KY 40292, USA; 2 Biostatistics and Bioinformatics Facility, James Graham Brown Cancer Center, University of Louisville School of Medicine, Louisville, KY 40292, USA; 3 Kentucky Biomedical Research Infrastructure Network Bioinformatics Core, University of Louisville School of Medicine, Louisville, KY 40202, USA; 4 Genomics Facility, University of Louisville School of Medicine, Louisville, KY 40292, USA; 5 Department of Medicine, James Graham Brown Cancer Center, University of Louisville School of Medicine, Louisville, KY 40292, USA

**Keywords:** melanoma, sentinel lymph node, recurrence, aging, Wnt signaling

## Abstract

Age is an important factor for determining the outcome of melanoma patients. Sentinel lymph node (SLN) status is also a strong predictor of survival for melanoma. Paradoxically, older melanoma patients have a lower incidence of SLN metastasis but a higher mortality rate when compared with their younger counterparts. The mechanisms that underlie this phenomenon remain unknown. This study uses three independent datasets of RNA samples from patients with melanoma metastatic to the SLN to identify age-related transcriptome changes in SLNs and their association with outcome. Microarray was applied to the first dataset of 97 melanoma patients. NanoString was performed in the second dataset to identify the specific immune genes and pathways that are associated with recurrence in younger versus older patients. qRT-PCR analysis was used in the third dataset of 36 samples to validate the differentially expressed genes (DEGs) from microarray and NanoString. These analyses show that FOS, NR4A, and ITGB1 genes were significantly higher in older melanoma patients with positive SLNs. IRAK3- and Wnt10b-related genes are the major pathways associated with recurrent melanoma in younger and older patients with tumor-positive SLNs, respectively. This study aims to elucidate age-related differences in SLNs in the presence of nodal metastasis.

## INTRODUCTION

Age is recognized as an important, yet complex, factor for determining the outcome of melanoma patients. Older melanoma patients with regional node metastases are known to have a worse prognosis than younger patients [1]; however, with the emergence of new treatments, more studies have found that older melanoma patients may have a better response to immunotherapy than their younger counterparts [2–4]. Most of these studies, though, only focus on the local immune environment in the primary tumor [2]. Few studies emphasize age-related transcriptome changes in the sentinel lymph node (SLN) and the association with outcome. This study aims to elucidate age-related differences in SLNs in the presence of nodal metastasis.

The SLN is the first lymph node that receives lymphatic drainage from the primary tumor. Lymph nodes are the sites where the adaptive immune response is mounted. SLN status is also the strongest predictor of survival for patients with clinically localized melanoma [5–7], but the majority of patients with melanoma metastasis to the SLN do not experience recurrence of their melanoma [8]. Paradoxically, older melanoma patients have a decreased risk of SLN metastasis but an increased risk of melanoma-specific mortality [9–11]. Clearly, there are some unidentified associations between SLN status and age in determining the outcome of melanoma patients. Therefore, the purpose of this study is to identify age-related transcriptome changes in the SLN from patients with melanoma metastasis to the SLN and to evaluate the relationship with melanoma recurrence. We used two different technologies (microarray and NanoString analysis) in three independent datasets of RNA samples obtained from melanoma patients with positive SLNs to identify age-related transcriptome changes in the SLN and their association with outcome. Our results show that FOS, NR4A, and ITGB1 genes were significantly higher in older melanoma patients with tumor-positive SLNs. Interleukin-1 receptor-associated kinase 3 (IRAK3) is the major pathway involved in recurrence in younger patients, while Wnt10b-related genes is the major pathway associated with recurrence in older patients with tumor-positive SLNs. Our current study may help to elucidate age-related differences in the response of the SLN to the presence of nodal metastasis.

## RESULTS

### Transcriptome changes in SLN genes in older patients (≥60 years old) versus younger patients (<60 years old) by microarray analysis

In our previous studies, we have found that expression of two SLN genes (PIGR and TFAP2A), when combined with clinicopathological features, correlated with prognosis in SLN-positive melanoma patients [12]. Since age is also an important factor in determining patient outcomes, we were interested in comparing gene expression profiles in older versus younger patients and in assessing whether there was a correlation with melanoma recurrence. Therefore, we analyzed the first microarray dataset from 97 melanoma patients with positive SLNs from the Sunbelt Melanoma Trial (SMT) and evaluated the transcriptome changes of the SLN by two defined age groups: the older and the younger groups. To ensure that we had a large enough sample size for a robust analysis, patients were defined as being older if they were ≥60 years old (yr^60+^). Patients were defined as being younger if they were <60 years old (yr^60-^).

Table 1 lists the clinical data of the 97 melanoma patients grouped by age. In this dataset, there were no significant differences between the two age groups in primary tumor site, Breslow thickness, Clark level, or ulceration presence. However, in younger patients, the recurrence rate was significantly higher when Breslow thickness was higher. In older patients, there were no significant differences in Breslow thickness, Clark level, and ulceration presence between groups of patients with recurrence (recur^yes^) and those without recurrence (recur^no^). Using microarray filter T3 and T4, we detected a total of 577 and 156 differentially expressed probe sets, in older versus the younger patients. Among them, there were 41 and 11 differentially expressed probe sets by filters T3 and T4 in the older versus younger groups (*p* <0.05). Probe sets without defined gene names by annotation from Partek Genomics Suite software were removed from the lists. There were 7 differentially expressed genes (DEGs) in the yr^60+^ group versus the yr^60-^ group with a *p* value < 0.05 by T4 filter (Table 2). Among them, FBJ murine osteosarcoma viral oncogene homolog (FOS) and nuclear receptor subfamily 4, group A, member 2 (NR4A2), were the two genes that had significant higher expression in the yr^60+^ group than in the yr^60-^ group. The DEGs between the yr^60-^ and the yr^60+^ group had various biological functions, including toll-like receptor signaling pathway transduction, adaptive and innate immune response, autophagy, and transcription regulation (Table 2). The network connection of the 156 DEGs by T4 filter is shown in Supplementary Figure 1. The top canonical pathway that showed a difference in the yr^60-^ and the yr^60+^ group was the peroxisome proliferator-activated receptor (PPAR) signaling pathway, which had a close interaction with toll-like receptor signaling pathway (Supplementary Table 1) [13, 14]. The list of all DEGs by T3 filter is listed in Supplementary Table 2.

**Table 1:** Clinical data of the first dataset (97 melanoma patients) grouped by age.

**Variables**	**Age ≤ 60**			**Age >60**			***P* value for age<60 vs age≥60**
	**No recurrence (N=51)**	**Recurrence (N=28)**	**P value**	**No recurrence (N=7)**	**Recurrence (N=11)**	***P* value**	
**Gender**			0.957			1.000 ^†^	0.541
Female (%)	24 (47.1)	13 (46.4)		3 (42.9)	4 (36.4)		
Male (%)	27 (52.9)	15 (53.6)		4 (57.1)	7 (63.6)		
**Primary Site**			0.908 ^†^			0.141 ^†^	0.371 ^†^
Head (%)	2 (3.9)	1 (3.6)					
Lower Extremity (%)	12 (23.5)	9 (32.1)		2 (28.6)	5 (45.5)		
Neck (%)	1 (2.0)	0 (0.0)		0 (0.0)	1 (9.1)		
Trunk (%)	28 (54.9)	13 (46.4)		4 (57.1)	2 (18.2)		
Upper Extremity (%)	8 (15.7)	5 (17.9)		0 (0.0)	3 (27.3)		
**Breslow Thickness (mm)**			***0.006***			0.837	0.362
Mean (95%CI)	2.5 (2.2 - 2.9)	3.9 (2.8 - 5.0)		2.6 (1.2 - 4.1)	2.5 (1.8 - 3.1)		
Median (min - max)	2.0 (1.0 - 6.0)	2.7 (1.5 - 13.0)		2.5 (1.2 - 6.8)	2.4 (1.1 - 4.4)		
**Clark level**			0.741 ^†^			1.000 ^†^	0.457 ^†^
II/III (%)	7 (13.7)	3 (10.7)		2 (28.6)	2 (18.2)		
IV/V (%)	43 (84.3)	25 (89.3)		5 (71.4)	9 (81.8)		
**Ulceration Present**			0.255 ^†^			0.430 ^†^	0.268 ^†^
NA (%)	0 (0.0)	1 (3.6)		1 (14.3)	0 (0.0)		
No (%)	34 (66.7)	15 (53.6)		3 (42.9)	5 (45.5)		
Yes (%)	17 (33.3)	12 (42.9)		2 (28.6)	6 (54.5)		
**Time To FU (All Patients)**			**<.001**			***0.015***	0.187
Mean (95%CI)	86.8 (80.8 - 92.7)	65.5 (53.3 - 77.7)		88.7 (73.0 - 104.5)	57.3 (42.3 - 72.2)		
Median (min - max)	92.0 (40.0 - 122.0)	58.5 (6.0 - 122.0)		94.0 (51.0 - 111.0)	57.0 (16.0 - 111.0)		

^†^ Fisher’s exact test.

**Table 2:** The DEGs in the SLN in yr^60+^ versus yr^60-^ patients in the microarray dataset using T4 filter (*P*<0.05).

**Gene symbol**	**Gene Name**	**Biological function**	***P* value**	**Fold change**
**FOSB**	FBJ murine osteosarcoma viral oncogene homolog B	negative regulation of transcription from RNA polymerase II promoter	0.021	1.60
**FOS**	FBJ murine osteosarcoma viral oncogene homolog	toll-like receptor signaling pathway//MyD88-dependent and -independent toll-like receptor signaling pathway	0.0255	1.56
**NR4A2**	nuclear receptor subfamily 4, group A, member 2	negative regulation of transcription from RNA polymerase II promoter//response to hypoxia	0.0096	1.47
**CLEC4C**	C-type lectin domain family 4, member C	stimulatory C-type lectin receptor signaling pathway//adaptive and innate immune response	0.049	1.45
**LIX1**	limb and CNS expressed 1	autophagy//autophagosome maturation	0.0098	1.41
**NRCAM**	neuronal cell adhesion molecule	angiogenesis//neuron migration/cell adhesion	0.0008	1.40
**GRB14**	growth factor receptor bound protein 14	signal transduction	0.0135	0.79

### Transcriptome changes of immune genes and immune pathway genes in the SLNs of older versus younger patients as assessed by NanoString analysis

Immune cells are a major component of lymph node structure. We then focused on immune genes and immune pathways associated with both age groups and assessed by NanoString analysis. This analysis in the second dataset found that 12 immune-related genes were differentially expressed in SLNs in older versus younger patients (Table 3). There were 17 immune pathway-related genes in SLNs that were differentially expressed in yr^60+^ versus yr^60-^ patients (Table 4). Of note is that the NR4A2 gene was found to be differentially expressed in yr^60+^ versus yr^60-^ patients from the first microarray dataset. The NR4A3 gene, which belongs to the same family members of NR4A2, was also found to have a higher fold change (FC) in yr^60+^ patients, even though the *p* value is borderline (*p*=0.0517) (last row in Table 4). The immune gene, integrin subunit beta 1 (ITGB1), was found to be differentially expressed in yr^60+^ versus the yr^60-^ patients (Table 3). Integrin subunit beta like 1 (ITGBL1) was also found to be differentially expressed in yr^60+^ versus yr^60-^ patients by microarray analysis (Supplementary Table 2). The immune gene with the highest and lowest fold change in the yr^60+^ versus the yr^60-^ patient group was melanoma antigen family A, 3 (MAGEA3) and leukemia inhibitory factor (LIF) (fold change =2.87 and -1.16) (Table 3). Among the three highest fold changes of the immune pathway genes, two of them were secreted frizzled-related protein 2 and 4 (SFRP2 and SFRP4) (fold change =1.93 and 1.78) (Table 4). Both genes belong to the Wnt pathway. A volcano plot of the immune genes and immune pathway genes that were differentially expressed in yr^60+^ versus the yr^60-^ patients is presented in Supplementary Figures 2, and 3.

**Table 3:** Immune genes that were differentially expressed in the SLN in yr^60+^ versus yr^60-^ patients in the second dataset by NanoString analysis (*P*<0.05).

**Gene symbol**	**Gene Name**	***P* value**	**Fold change**
**MAGEA3**	melanoma antigen family A, 3	0.0149	2.87
**MME**	membrane metallo-endopeptidase	0.0466	1.34
**CD244**	CD244 molecule, natural killer cell receptor 2B4	0.0453	1.07
**CDKN1A**	cyclin-dependent kinase inhibitor 1A (p21, Cip1)	0.0254	0.792
**JAM3**	junctional adhesion molecule 3	0.0405	0.628
**ITGB1**	integrin subunit beta 1	0.00565	0.564
**ALCAM**	activated leukocyte cell adhesion molecule	0.00272	0.489
**MAVS**	mitochondrial antiviral signaling protein	0.00886	0.303
**IFIH1**	interferon induced with helicase C domain 1	0.0118	-0.325
**MX1**	myxovirus (influenza virus) resistance 1, interferon-inducible protein p78 (mouse)	0.0493	-0.666
**CXCL3**	chemokine (C-X-C motif) ligand 3	0.0393	-0.851
**LIF**	leukemia inhibitory factor	0.0476	-1.16

**Table 4:** Immune pathway genes that are differentially expressed in the SLN in yr^60+^ versus yr^60-^ patients in the second dataset by NanoString analysis (*P*<0.05)*.

**Gene symbol**	**Gene Name**	***P* value**	**Fold change**
**COMP**	cartilage oligomeric matrix protein	0.0212	2.51
**SFRP2**	secreted frizzled-related protein 2	0.0428	1.93
**SFRP4**	secreted frizzled-related protein 4	0.0279	1.78
**CTNNB1**	catenin (cadherin-associated protein), beta 1, 88kDa	0.0247	0.832
**CDKN1A**	cyclin-dependent kinase inhibitor 1A (p21, Cip1)	0.0345	0.702
**PLD1**	phospholipase D1, phosphatidylcholine-specific	0.0088	0.661
**TNC**	tenascin C	0.0207	0.655
**PBRM1**	polybromo 1	0.00195	0.463
**GADD45A**	growth arrest and DNA-damage-inducible, alpha	0.00483	0.421
**FUT8**	fucosyltransferase 8 (alpha (1,6) fucosyltransferase)	0.0394	0.367
**PIK3R3**	phosphoinositide-3-kinase, regulatory subunit 3 (gamma)	0.0278	0.317
**PRKAR2A**	protein kinase, cAMP-dependent, regulatory, type II, alpha	0.0167	0.296
**PIK3CB**	phosphatidylinositol-4,5-bisphosphate 3-kinase, catalytic subunit beta	0.0454	0.283
**FANCB**	Fanconi anemia, complementation group B	0.0385	-0.589
**ERCC2**	excision repair cross-complementing rodent repair deficiency, complementation group 2	0.0176	-0.595
**TNFRSF10C**	tumor necrosis factor receptor superfamily, member 10c, decoy without an intracellular domain	0.0123	-0.677
**CDK6**	cyclin-dependent kinase 6	0.0308	-0.683
**NR4A3**	nuclear receptor subfamily 4, group A, member 3	0.0517	1.3

*Except the last row of NR4A3 gene has a p-value of 0.0517.

NanoString results suggested that NR4A and ITGB1 genes are highly expressed immune genes in older melanoma patients rather than their younger counterparts with lymph node metastasis. These genes, therefore, might be responsible for the age-related differences in response of SLN to the presence of nodal metastasis. The Wnt pathway might also be an important immune pathway associated with age-related immune response to melanoma metastasis to the SLN.

### Transcriptome changes in SLN associated with recurrence in yr^60+^ or yr^60-^ melanoma patients by microarray analysis

After we compared the transcriptome changes in SLN genes between the yr^60+^ and the yr^60-^ melanoma patients, we studied whether there were any differences between patients who experienced recurrence versus those who remained disease free. We also evaluated these results by age categories. A multivariable linear regression model was fitted for each gene of each sample about age (yr^60+^ or yr^60-^), outcome (recur^yes^ or recur^no^), and the interaction of age and outcome in the first microarray dataset. There were 100 differentially expressed probe sets with a statistically significant difference (*p* <0.05) after adjusting either by age or outcome or the interaction of age and outcome using filter T4. (Data not listed due to the long list of genes, but available upon request.) Among them, there were 11 differentially expressed probe sets with a significant difference adjusting by the interaction of age and outcome (*p* <0.05). Probe sets of the same gene were merged. There were 6 genes with statistically significant differences between groups (Table 5). We further analyzed the mean and 95% confidence interval (CI) of these 6 DEGs (Table 5). Means (95% CI) without overlapped values between each group were italicized. The non-overlapped values implied that there were statistically significant differences between the two groups. For example, NR4A2 was differentially expressed in yr^60+^ versus yr^60-^ melanoma patients without recurrence. NR4A2 also showed significant differences in yr^60+^ patients with (recur^yes^) versus those without recurrence (recur^no^) (Table 5).

**Table 5:** Mean and 95% confidence interval (CI) of the DEGs adjusted by the interaction of age and outcome using a multivariable linear regression model in the first microarray dataset.

**Variables**	**Total (N=97)**	**Young (<60 years old)**		**Old (≥ 60 years old)**		**P value**
		**No recurrence (N=51)**	**Recurrence (N=28)**	**No recurrence (N=7)**	**Recurrence (N=11)**
**NR4A2**						<.001
Mean (95% CI)	6.0 (5.8 - 6.2)	*6.0 (5.8 - 6.2)*	5.6 (5.5 - 5.8)	*7.6 (6.4 - 8.8)*	*5.7 (5.5 - 6.0)*	
Mean ± SE	6.0 ± 0.1	6.0 ± 0.1	5.6 ± 0.1	7.6 ± 0.6	5.7 ± 0.1	
**IL1B**						<.001
Mean (95% CI)	6.5 (6.3 - 6.6)	*6.5 (6.3 - 6.7)*	6.2 (5.9 - 6.5)	*7.8 (6.6 - 9.0)*	*6.0 (5.7 - 6.2)*	
Mean ± SE	6.5 ± 0.1	6.5 ± 0.1	6.2 ± 0.1	7.8 ± 0.6	6.0 ± 0.1	
**TFPI2**						<.001
Mean (95%CI)	5.5 (5.4 - 5.7)	*5.7 (5.5 - 5.9)*	*5.4 (5.1 - 5.8)*	6.4 (5.8 - 7.0)	*4.7 (4.2 - 5.1)*	
Mean ± SE	5.5 ± 0.1	5.7 ± 0.1	5.4 ± 0.2	6.4 ± 0.3	4.7 ± 0.2	
**CLEC7A**						0.004
Mean (95%CI)	4.7 (4.5 - 4.9)	*4.8 (4.6 - 5.1)*	*4.6 (4.3 - 4.9)*	*5.2 (4.4 - 6.0)*	*3.9 (3.5 - 4.3)*	
Mean ± SE	4.7 ± 0.1	4.8 ± 0.1	4.6 ± 0.2	5.2 ± 0.4	3.9 ± 0.2	
**PTGS2**						0.001
Mean (95% CI)	6.1 (5.8 - 6.3)	6.2 (5.9 - 6.5)	*5.7 (5.3 - 6.1)*	*7.6 (6.2 - 9.0)*	5.5 (4.8 - 6.3)	
Mean ± SE	6.1 ± 0.1	6.2 ± 0.2	5.7 ± 0.2	7.6 ± 0.7	5.5 ± 0.4	
**RGS1**						<.001
Mean (95% CI)	5.9 (5.7 - 6.2)	*6.2 (5.9 - 6.5)*	*5.5 (5.1 - 5.9)*	*7.2 (6.2 - 8.2)*	*5.1 (4.4 - 5.8)*	
Mean ± SE	5.9 ± 0.1	6.2 ± 0.1	5.5 ± 0.2	7.2 ± 0.5	5.1 ± 0.4	

### Transcriptome changes of immune genes and immune pathway genes in SLNs associated with recurrence in yr^60-^ and yr^60+^ melanoma patients by NanoString analysis

In the NanoString dataset, we first analyzed the differentially expressed immune genes between recur^yes^ and recur^no^ groups in younger melanoma patients (yr^60-^). The results showed that there were 20 differentially expressed immune genes (*p*<0.05) in this comparison (Supplementary Table 3). Selected differentially expressed immune genes between the recur^yes^ and recur^no^ patients with *p*<0.05 and absolute fold change >0.5 in the yr^60-^ group were listed in Table 6. In yr^60-^ patients with positive SLNs, highly expressed C6, interleukin 23 receptor (IL23R), B melanoma antigen (BAGE), chemokine [C-C motif] ligand 16 (CCL16), and lower expression of S100 calcium binding protein B (S100B) were associated with recur^yes^ patients. A volcano plot of the immune genes that were differentially expressed in younger patients (yr^60-^) in the recur^yes^ versus the recur^no^ groups is shown in Supplementary Figure 4.

**Table 6:** Selected differentially expressed immune genes between recur^yes^ and recur^no^ group in younger patients (yr^60-^) by NanoString analysis (*p*<0.05, absolute fold change > 0.5).

**Gene symbol**	**Gene Name**	***P* value**	**Fold change**
**C6**	complement component 6	0.00745	4.28
**IL23R**	interleukin 23 receptor	0.00545	3.64
**BAGE**	B melanoma antigen	0.0136	3.58
**CCL16**	chemokine (C-C motif) ligand 16	0.0168	3.46
**SPINK5**	serine peptidase inhibitor, Kazal type 5	0.0161	2.96
**MAPK11**	mitogen-activated protein kinase 11	0.00968	2.84
**MST1R**	macrophage stimulating 1 receptor (c-met-related tyrosine kinase)	0.00947	2.52
**F2RL1**	coagulation factor II (thrombin) receptor-like 1	0.00399	1.97
**DOCK9**	dedicator of cytokinesis 9	0.00847	1.61
**IGF1R**	insulin-like growth factor 1 receptor	0.0141	0.971
**TBK1**	TANK-binding kinase 1	0.0116	0.616
**ICAM1**	intercellular adhesion molecule 1	0.0143	-0.587
**C1QBP**	complement component 1, q subcomponent binding protein	0.0131	-0.9
**PSMB8**	proteasome (prosome, macropain) subunit, beta type, 8 (large multifunctional peptidase 7)	0.0121	-0.939
**MIF**	macrophage migration inhibitory factor (glycosylation-inhibiting factor)	0.016	-1.09
**HLA-G**	major histocompatibility complex, class I, G	0.00237	-1.18
**S100B**	S100 calcium binding protein B	0.00859	-5.64

In older patients, there were 20 differentially expressed genes between the recur^yes^ and recur^no^ group (*p*<0.05) (Supplementary Table 4). Table 7 lists the selected differentially expressed immune genes by recurrence status in the yr^60+^ melanoma patients with *p*<0.05 and absolute fold change > 0.5. In yr^60+^ patients with positive SLNs, highly expressed FOS and CCL18 were associated with recur^yes^. A volcano plot of the immune genes that was differentially expressed in older patients in the recur^yes^ versus the recur^no^ group is shown in Supplementary Figure 5.

**Table 7:** Selected differentially expressed immune genes between the recur^yes^ and recur^no^ group in yr^60+^ patients by NanoString analysis (*p*<0.05, absolute fold change > 0.5).

**Gene symbol**	**Gene Name**	***P* value**	**Fold change**
**FOS**	FBJ murine osteosarcoma viral oncogene homolog	0.0221	1.9
**CCL18**	chemokine (C-C motif) ligand 18 (pulmonary and activation-regulated)	0.00867	1.8
**CXCR4**	chemokine (C-X-C motif) receptor 4	0.0238	1.07
**C3**	complement component 3	0.00481	0.832
**TLR10**	toll-like receptor 10	0.0189	0.787
**NOD1**	nucleotide-binding oligomerization domain containing 1	0.00347	0.768
**PLAU**	plasminogen activator, urokinase	0.00371	0.741
**CYBB**	cytochrome b-245, beta polypeptide	0.00314	0.732
**TLR6**	toll-like receptor 6	0.013	0.626
**HLA-DMA**	major histocompatibility complex, class II, DM alpha	0.0192	0.606
**TNFRSF13B**	tumor necrosis factor receptor superfamily, member 13B	0.0191	0.555
**CD84**	CD84 molecule	0.014	0.504
**RELA**	v-rel reticuloendotheliosis viral oncogene homolog A (avian)	0.0225	-0.52
**IFITM1**	interferon induced transmembrane protein 1	0.0176	-0.675
**NCAM1**	neural cell adhesion molecule 1	0.00896	-0.984

When comparing the difference in the DEGs by recurrence status in both age groups, we found that MAPK11 was highly expressed in the younger melanoma patients in the recur^yes^ versus the recur^no^ group (FC=2.84) (Table 6). A similar family member, MAP2K4, had marginal expression in older patients in the recur^yes^ versus the recur^no^ group (FC=0.25) (Supplementary Table 4). CCL16 had a higher expression in the younger patient cohort in the recur^yes^ versus the recur^no^ group (FC=3.46) (Table 6). Another family member, CCL18, also had a higher expression in older patients with recurrence (FC=1.8) (Table 7). C6 was highly expressed in younger melanoma patients with recurrence (FC=4.28) (Table 6), while C3 had marginal expression in older patients with recurrence (FC=0.83) (Table 7).

In terms of immune pathway genes, there were 18 differentially expressed genes with *p*<0.05 and absolute fold change > 0.5 in the younger patients when comparing recur^yes^ versus recur^no^ (Table 8). A complete list of the DEGs with *p*<0.05 is presented in Supplementary Table 5. Supplementary Figure 6 shows a volcano plot of the immune pathway genes in younger patients that were differentially expressed by recurrence status. In the group of older patients, there were 13 differentially expressed immune pathway genes with *p*<0.05 and absolute fold change > 0.5 by recurrence status (Table 9). Supplementary Figure 7 shows a volcano plot of the immune pathway genes that were differentially expressed in older patients based on recurrence status. All the DEGs with *p*<0.05 in the older group are listed in Supplementary Table 6. IRAK3 (interleukin-1 receptor-associated kinase 3) was the major immune pathway gene found in younger patients with recurrence (Table 8), while Wnt10b was the major pathway found in older patients with recurrence (Table 9). There were no overlapped immune pathway genes in either age group by recurrence status. These results suggested that, even though some immune genes have similar changes in older and younger patients, different pathways may be involved in recurrence in different age groups.

**Table 8:** Differentially expressed immune pathway genes in younger patients (yr^60-^) between the recur^yes^ and recur^no^ group by NanoString analysis (*p*<0.05, absolute fold change > 0.5).

**Gene symbol**	**Gene Name**	***P* value**	**Fold change**
**IRAK3**	interleukin-1 receptor-associated kinase 3	0.00552	2.15
**NKD1**	naked cuticle homolog 1 (Drosophila)	0.00565	2.13
**ACVR1C**	activin A receptor, type IC	0.0111	1.9
**SOS1**	son of sevenless homolog 1 (Drosophila)	0.00681	1.42
**EPOR**	erythropoietin receptor	0.00773	1.32
**ACVR2A**	activin A receptor, type IIA	0.012	1.12
**RAD50**	RAD50 homolog (S. cerevisiae)	0.0101	0.83
**SMAD2**	SMAD family member 2	0.00233	0.799
**DNMT3A**	DNA (cytosine-5-)-methyltransferase 3 alpha	0.0113	0.745
**RPS6KA5**	ribosomal protein S6 kinase, 90kDa, polypeptide 5	0.00593	0.596
**FANCL**	Fanconi anemia, complementation group L	0.00732	-0.554
**PPP2R1A**	protein phosphatase 2, regulatory subunit A, alpha	0.00791	-0.679
**RB1**	retinoblastoma 1	0.0108	-0.84
**UBB**	ubiquitin B	0.0109	-0.849
**CDK4**	cyclin-dependent kinase 4	0.00456	-1.22
**CASP9**	caspase 9, apoptosis-related cysteine peptidase	0.00969	-1.22
**HSP90B1**	heat shock protein 90kDa beta (Grp94), member 1	0.0106	-1.25
**PCNA**	proliferating cell nuclear antigen	0.00314	-1.45

**Table 9:** Differentially expressed immune pathway genes in older patients (yr^60+^) between the recur^yes^ and recur^no^ group by NanoString analysis (*p*<0.05, absolute fold change > 0.5).

**Gene symbol**	**Gene Name**	***P* value**	**Fold change**
**WNT10B**	wingless-type MMTV integration site family, member 10B	0.027	2.27
**HSPA1A**	heat shock 70kDa protein 1A	0.0283	2.04
**FOS**	FBJ murine osteosarcoma viral oncogene homolog	0.0219	1.96
**DKK2**	dickkopf WNT signaling pathway inhibitor 2	0.0247	1.7
**IL6**	interleukin 6 (interferon, beta 2)	0.00111	1.36
**TGFB3**	transforming growth factor, beta 3	0.0379	1.19
**HHEX**	hematopoietically expressed homeobox	0.0263	1.06
**DLL4**	delta-like 4 (Drosophila)	0.00752	0.883
**XRCC4**	X-ray repair complementing defective repair in Chinese hamster cells 4	0.0354	0.787
**NR4A1**	nuclear receptor subfamily 4, group A, member 1	0.0232	0.766
**ALKBH2**	alkB, alkylation repair homolog 2 (E. coli)	0.0384	0.752
**PLAU**	plasminogen activator, urokinase	0.00195	0.659
**BID**	BH3 interacting domain death agonist	0.0369	0.564

### Verification of the DEGs in the third independent dataset by quantitative reverse transcriptase polymerase chain reaction (qRT-PCR)

**Figure 1:**
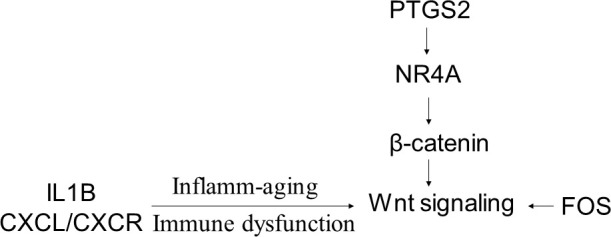
**A schematic model showing that the DEGs in the older melanoma patients by recurrence status converge at the Wnt signaling pathway.**

After using microarray and NanoString technologies to identify the DEGs in the yr^60-^ and yr^60+^ patients as well as the different outcome-associated age groups (recur^yes^ versus recur^no^), we used qRT-PCR in another independent dataset to confirm the findings above. We selected genes that were differentially expressed in both microarray and NanoString analysis or had higher fold changes in either of the analysis. The results showed that FOS, NR4A2, PTGS2, LINC00518, IL1B, and Wnt10b were all highly expressed in older patients with recurrence (Table 10). These genes converged at the Wnt10b pathway (Figure 1).

**Table 10:** qRT-PCR validation of the DEGs in the third independent dataset (recur^yes^ versus recur^no^).

**Gene name**	**Fold change**	
	**Age <60**	**Age ≥ 60**
FOS	+1.2	+12.6
NR4A2	+1.4	+4.2
PTGS2	+1.8	+3.5
LINC00518	+3.2	+9.0
IL1B	-1.2	+1.1
Wnt10b	+1.6	+2.9
CCL18	-1.7	+1.3
HSPA1A	-1.2	+1.3
NRCAM	+1.14	+2.0
CXCL5	+1.4	+1.6

## DISCUSSION

Older melanoma patients have specific prognostic features that distinguish them from younger patients. Older melanoma patients generally have melanomas with greater Breslow thickness [10, 15, 16]. The incidence of ulceration and regression is also increased with age [10]. On the other hand, the incidence of SLN metastasis declines with increasing age [10]. Few studies have shed light on the impact of age on melanoma prognosis. Even fewer studies have connected the genetic changes with the outcomes associated with increasing age in melanoma patients. In this current study, we used three independent datasets and two different technologies, microarray and NanoString, to identify the DEGs in SLNs that are associated with recurrence by age group. NanoString used a novel method of direct mRNA barcoding and digital detection of target molecules through the use of color-coded probe pairs. This new technology does not need reverse transcription and the downstream PCR amplification to assess the gene expression level. We selected an immune panel and an immune pathway panel for NanoString analysis to focus on immune-related gene changes in SLNs. The results showed that there was some overlap of DEGs (NR4A and FOS) that have been detected by both technologies. Those genes have been confirmed by PCR in an independent dataset. Some genes (PTGS2, IL1B, LINC00518, and Wnt10b) that have higher fold changes detected by either of the two technologies were also confirmed by PCR in an independent dataset. The two technologies complement each other. In combination with the three independent datasets used in this study, these data provide a higher standard of research integrity.

Our results showed that Wnt signaling and related genes in SLNs have significant changes that correlate with recurrence in older melanoma patients with SLN metastasis. The Wnt signaling pathway includes the canonical or β-catenin dependent pathway and the non-canonical or β-catenin independent pathway [17]. The two pathways ultimately converge. The canonical pathway is more characterized, with β-catenin as the primary effector. The non-canonical pathway includes the Ca^2+^/PKC pathway and the planar cell polarity pathway mediated via JNK signaling [18]. Recent studies have shown that the Wnt signaling has an important role during organism aging and may eventually affect age-related melanoma outcome through several different ways [18]. First, Wnt signaling can mediate age-related therapy resistant in melanoma through klotho. Klotho is an age-associated protein in which level decreases by age 40. Klotho can inhibit internalization and signaling of Wnt5A, which drives melanoma metastasis and resistance to targeted therapy [19]. Increasing klotho levels may improve the therapeutic effect of BRAF inhibitors by targeting Wnt signaling in melanoma patients of advanced age [19]. Second, Wnt effector, β-catenin, can interact with telomerase reverse transcriptase (TERT) to control age-related melanoma progression. TERT can directly bind to the β-catenin promoter region and activate its transcriptional activity [20, 21]. Loss of β-catenin increases telomere damage by triggering formation of telomere dysfunction-induced foci and causing the cells to enter senescence [21]. Third, Wnt regulates skin aging and modulates stromal microenvironment of the skin to affect tumor progression [18, 22]. Aged dermal fibroblasts secrete more of the Wnt antagonist SFRP2 to suppress β-catenin in melanoma cells and drive melanoma metastasis [22]. The repressed Wnt signaling makes melanoma cells more sensitive to oxidative stress and drive resistance to BRAF inhibitors [22]. Through Wnt signaling, therefore, the aging microenvironment in older melanoma patients drives more aggressive melanoma cell behavior and causes worse prognosis. In agreement with the results by Virós et al [22], we also found that SFRP2 and SFRP4 were among the highest fold change genes in older melanoma patients compared with their younger counterparts. Wnt signaling molecules and inhibitors are subtly balanced during aging to affect the melanoma outcome in older patients.

PTGS2-NR4A-Wnt forms a network to regulate tumor immunity. It was reported that inhibition of PTGS2 (also named COX2) correlates with the decreased expression of NR4A transcription factors and Treg genes in leukemia [23]. NR4A transcription factors can regulate β-catenin at the transcription level. The upregulated NR4A-Wnt signaling axis may act to attenuate anti-leukemia immunity by blocking the production of leukemia-reactive CD8 cytotoxic T lymphocytes [23]. Currently, no reports have shown how the PTGS2-NR4A-Wnt is associated with age-related immunity in melanoma. In our study, we found that Wnt10b was upregulated in older melanoma patients who experience recurrence. The upstream genes of PTGS2 and NR4A were also upregulated. How the PTGS2-NR4A-Wnt signaling coordinate together to connect with recurrence and immunity in the older melanoma patients needs to be further explored.

FOS gene and Wnt family member 7B (Wnt7B) were found to be enriched in the Wnt signaling pathway in gastric cancer [24]. Targeting on FOS and Wnt signaling can inhibit gastric cancer proliferation [24]. In colon cancer cells, suppressing FOS and β-catenin can effectively inhibit proliferation of cancer cells [25]. This provides an attractive strategy for the treatment of colon cancer by targeting the Wnt/ β-catenin signaling. FOS has been shown to be involved in cell proliferation, migration, and invasion accompanied by altered expression of Wnt family members in osteosarcoma [26]. Our results showed that FOS was the gene with the highest fold change in recur^yes^ versus recur^no^ in older melanoma patients. Upregulated FOS might occur in conjunction with activated Wnt pathway to promote melanoma progression in older patients.

Age is a complex factor in determining the outcome of melanoma patients [4]. Researchers have generally accepted that age is an independent prognostic factor for melanoma overall survival [10, 15, 27], particularly since the mortality rate of melanoma increases with age [27]. One reason that older patients have a higher mortality rate is that they tend to have thicker and more ulcerated melanomas than younger individuals [10, 15, 16]. Another reason may be attributed to the age- related immune dysfunction [28, 29]. Immune cell switching, inflamm-aging, and aged microenvironment, all drive age-induced tumor progression [28–30]. Inflamm-aging is defined as aging associated with a chronic, sterile, low-grade inflammation [31]. There is a direct influence of aging on immune cell type specificity and function within systemic and local microenvironments. Increased level of IL-1, IL-6, IL-1α, and IL-1β drives inflamm-aging response and contributes to mortalities in the older patient [29, 31]. CXCL/CXCR plays an important role in driving cell senescence. This may explain why there are numerous dysregulated immune genes associated with recurrence in older patients. How all these factors intertwine to determine the patients’ outcome needs further investigation.

We want to note that we did not stratify the effect of age on the treatment responses in this study. Our microarray dataset is from node-positive melanoma patients from the Sunbelt Melanoma Trial (SMT). This trial was from an era before modern adjuvant immunotherapy and targeted therapy. The patients were randomized to receive or not receive adjuvant interferon alfa-2b. The study showed that there is no improvement in recurrence-free or overall survival for interferon-treated patients [32]. The SLNs were all removed prior to therapy. Therefore, we do not believe that adjuvant therapy is likely to have biased results of this study in terms of the risk of recurrence. With the emergence of immunotherapy, the impact of age on the response to immunotherapy remains a hot topic. Most recent studies found that immune checkpoint inhibitors are surprisingly effective in older patients [2–4]. One possibility for this favored response in older people is that they may have a larger number of melanoma-reactive T cells than their younger counterparts [33]. Another possibility is changed T-cell populations in the elderly. CD8+ T cells are the primary target cells of checkpoint inhibitors. Decreased CD8+ cells, increased T regulatory cells (Tregs), and subsequent decreased CD8:FoxP3 ratio are more commonly found in the local tumor microenvironment of younger melanoma patients than older patients [2, 34, 35]. Depletion of Tregs can increase the response to immunotherapy in young mice [2]. This may explain why changes in the T-cell population have favored older patients in response to immune therapy. We are collecting SLN samples from melanoma patients by targeted therapy and immunotherapy in the hope of finding the difference in immune landscape of the SLN in response to different treatment regimens in younger versus older patients.

There are some limitations of our study. First is the sample size. For the microarray experiment, we randomly selected 97 SLNs from 317 patients with node-positive samples in the SMT. Analysis of the full enrollment from the SMT and other large datasets showed that as age increases, so does Breslow thickness [10, 27, 32]. In a different retrospective cohort study on 1931 cases of invasive melanoma, recurrence also was more likely to occur with increasing age [36]. In our current microarray dataset, there was no difference in recurrence between the younger and the older group (Table 1). Neither were there any differences in Breslow thickness in the younger versus older group because of small sample size and sample selection. We expect that if we had a larger sample size with significant difference in recurrence and Breslow thickness in the younger versus older group, we may have seen more significant differences of the differentially expressed genes between the groups. Nonetheless, the significant genes identified in this study may provide us with a starting point to further investigate and understand the age-related transcriptome landscape in SLN during melanoma recurrence. Our study focused on the genetic changes of SLN in response to the presence of nodal metastasis and their associations with age and outcome, but we have not compared the data of the primary tumor burden and the immune profile of the primary tumor with the genetic changes of SLN associated with age. Immune cells and tumor cells traffic between the primary tumor and lymph node. The tumor-lymph node axis acts systemically in controlling the patients’ outcome. Combining the data of the primary tumor with SLNs may provide a complete picture of the patients’ genetic profile to better guide treatment strategy. We also want to note that the genetic changes in SLN in node-negative patients may be different from those of the node-positive patients. We are planning to investigate those changes in the near future.

## CONCLUSIONS

The Wnt pathway, specifically Wnt10b, is a major pathway associated with melanoma recurrence in older patients with tumor-positive SLNs. These findings may lead to better understanding of the genetic changes associated with different outcome and develop new therapeutic strategies for older patients. Further research is ongoing to define the mechanisms by which Wnt signaling and related genes may predispose older patients to poor prognosis.

## MATERIALS AND METHODS

### Patient selection

This study used two different technologies in three independent datasets of RNA samples obtained from melanoma patients with positive SLNs to identify age-related transcriptome changes in SLN and their association with outcome.

Microarray analysis was performed in the first independent dataset to assess 97 samples obtained from the Sunbelt Melanoma Trial (SMT). The samples were randomly chosen from among 317 melanoma patients with positive SLNs. This patient cohort has been described previously [12]. Thirty-nine patients experienced recurrence melanoma in this cohort, and fifty-eight patients did not experience recurrence. Median follow-up was 93 months [12]. This study was approved by the institutional review boards (IRB) of each participating institution. Clinicopathologic factors, recurrence, and survival data were collected prospectively. Additional details of the SMT are described elsewhere [12, 32].

NanoString analysis was applied to the second patient cohort, which included 12 patients with tumor-positive SLNs from the James Graham Brown Cancer Center Biorepository at University of Louisville. This study followed an approved IRB protocol. There were 6 patients who experienced recurrence (3 of each at age <60 and ≥ 60 years old) and 6 patients who did not experience recurrence (3 of each at age <60 and ≥ 60 years old). Median follow-up was 34 months.

The third independent dataset of 36 samples from the James Graham Brown Cancer Center Biorepository was used to validate the differentially expressed genes (DEGs). The SLN tissue was acquired from patients at the time of surgical treatment of cutaneous melanoma, including staging with SLN biopsy between 2003 and 2017. Median follow-up of this cohort was 33.2 months. Patient characteristics such as age and outcome from all three datasets are summarized in Supplementary Table 7.

### Definition of age groups

To ensure that we had a large enough sample size for a robust analysis, we grouped patients into two age groups. Patients were defined as being older if they were ≥60 years old (yr^60+^). Patients were defined as being younger if they were <60 years old (yr^60-^).

### Microarray experiments

GeneChip Human HG-U133 plus 2.0 array (Affymetrix, Santa Clara, CA) was used in the first microarray dataset according to the manufacturer’s guidelines. Details of RNA isolation, microarray experiment, and quality control were described in detail previously [12]. This set of microarray data is accessible through NCBI’s Gene Expression Omnibus (GEO, http://www.ncbi.nlm.nih.gov/geo) by accession number GSE 43081.

### NanoString analysis of mRNA expression of immune panel genes and immune pathway panel genes

The second dataset of 12 RNA samples were isolated from fresh-frozen human SLN tissues from melanoma patients using RNeasy Plus Mini Kit (Qiagen). RNA quality control/quantity assessment (QC/QA) was checked by Agilent bioanalyzer. The RNA concentration was measured by Qubit. Total RNA (100 ng per sample) were analyzed on the nCounter MAX system. Two gene expression assays were used: PanCancer immune profiling and PanCancer immune pathway profiling (NanoString Technologies, Seattle, WA, USA). PanCancer immune profiling assay comprised 730 immune-related genes and 40 internal reference genes. Immune pathway profiling assay comprised 730 genes from 13 canonical pathways and 40 selected reference genes. Raw counts for each assay were collected using the NanoString data analysis software (nSolver).

### Quantitative reverse transcriptase polymerase chain reaction (qRT-PCR)

The third dataset of 36 RNA samples were isolated from fresh-frozen human SLN tissues from melanoma patients using RNeasy Plus Mini Kit (Qiagen). Total SLN RNA (1000 ng) from each sample was reverse-transcribed with the SuperScript III First-Strand Synthesis System. mRNA primers were purchased from Life Technologies (Carlsbad, CA). Quantitative RT-PCR reactions were completed on a 7500 Fast Real Time PCR system (Life Technologies). The relative quantity of the target mRNA was normalized to endogenous gene (B_2_M). The fold changes (FC) of each mRNA in the qRT-PCR experiments were calculated with the 2^-ΔΔCt^ method.

### Statistical analysis

For microarray analysis, a fold change outlier (FCO) filter was applied independently to reduce the dimension of the data before determining the DEGs between the two age groups (yr^60+^ and yr^60-^) as well as between patients with recurrence (recur^yes^) and those without recurrence (recur^no^) [37, 38]. For each of 54,675 probes on the array, the fold change (FC) was calculated and four filters (T1, T2, T3 and T4) were used. T1={μ(FC) ± 1.5σ(FC)}, T2={μ(FC) ± 2σ(FC)}, T3={μ(FC) ± 3σ(FC)}, and T4={μ(FC) ± 4σ(FC)}, where μ(FC) is the mean of fold changes (FC) and σ is the standard deviation of FC from all 54,675 probes in the array. The genes that fell inside T1, T2, and T3 were filtered from the differential data. After filtering the data, a t-test for normal gene expression data and a Wilcoxon test for non-normal expression data were applied [39]. The Benjamini-Hochberg method [40] was employed to adjust the *p* values. When comparing the changes of the SLN gene expressions in the yr^60+^ versus yr^60-^ patients, a multivariable linear regression model was fitted for each gene about age (<60 or ≥ 60). The equation used is below:

Gene Expression = α + β1 age, where age=1 if ≥60 years old and 0 otherwise.

The estimates and *p* values are presented by filter T2, T3, and T4. When assessing the changes of the SLN gene expressions that are associated with recur^yes^ versus recur^no^ in the yr^60+^ and yr^60-^ melanoma patients, a multivariable linear regression model was fitted for each gene of each sample about age (<60 years or ≥60), outcome (recur^yes^ or recur^no^), and the interaction of age and outcome. The equation used is below:

Gene Expression = α + β1 age+ β2 outcome+ β3 age*outcome, where age=1 if ≥60 years old and 0 otherwise, outcome=1 if recur^yes^ and 0 otherwise.

The estimate and *p* values are also presented by filter T2, T3, and T4. Statistical Analysis System (SAS) was used to perform the regression analysis [41, 42]. *p* values of FC were calculated using ANOVA.

For the NanoString results analysis, positive control normalization was performed by using gene expression data normalized to the mean of the positive control probes for each assay. RNA content normalization was performed by using gene expression data normalized to the geometric mean of housekeeping genes in the CodeSet. Raw data are also analyzed using the nSolver Advanced Analysis module. More information on the Advanced Analysis package can be found at http://www.nanostring.com/products/nSolver.

Ingenuity Pathway Analysis (IPA) software (Ingenuity Systems, Redwood City, CA) was used for gene network and pathway analysis. The statistical score of a pathway is defined as –log (*p* value) from Fisher’s exact test analysis.

## Supplementary Material

Supplementary Figures

Supplementary Tables
