# Supplementary material for: Age-related transcriptome changes in melanoma patients with tumor-positive sentinel lymph nodes
Source: Aging (Albany NY). 2020 Dec 29;12(24):24914–39. doi: 10.18632/aging.202435 (PMC7803563; doi:10.18632/aging.202435)
Supplement: Supplementary Figures [file aging-12-202435-s001.pdf]

SUPPLEMENTARY FIGURES

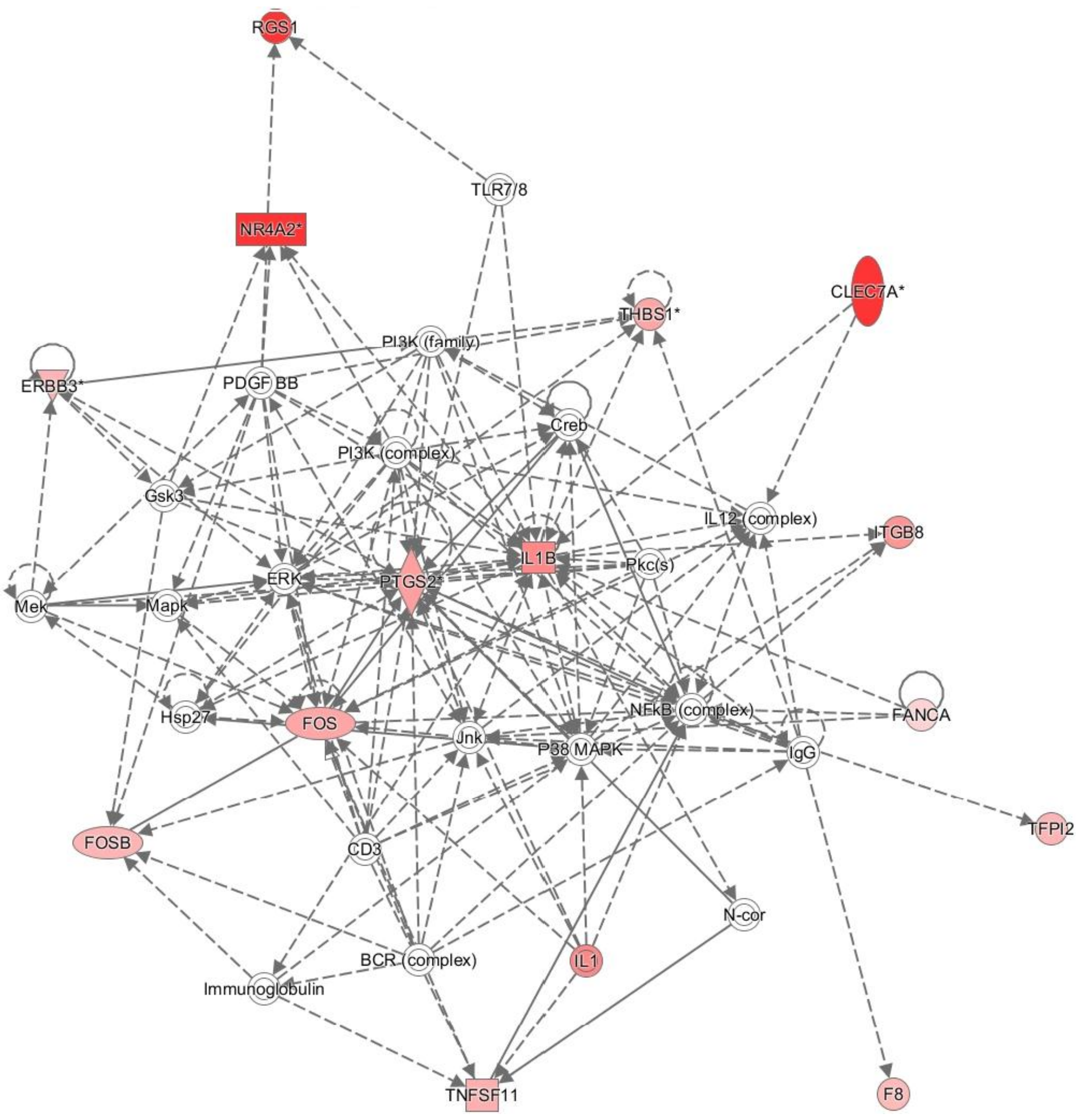

Supplementary Figure 1. The network connection of the 156 DEGs in the older versus the younger patients by microarray T4 filter.

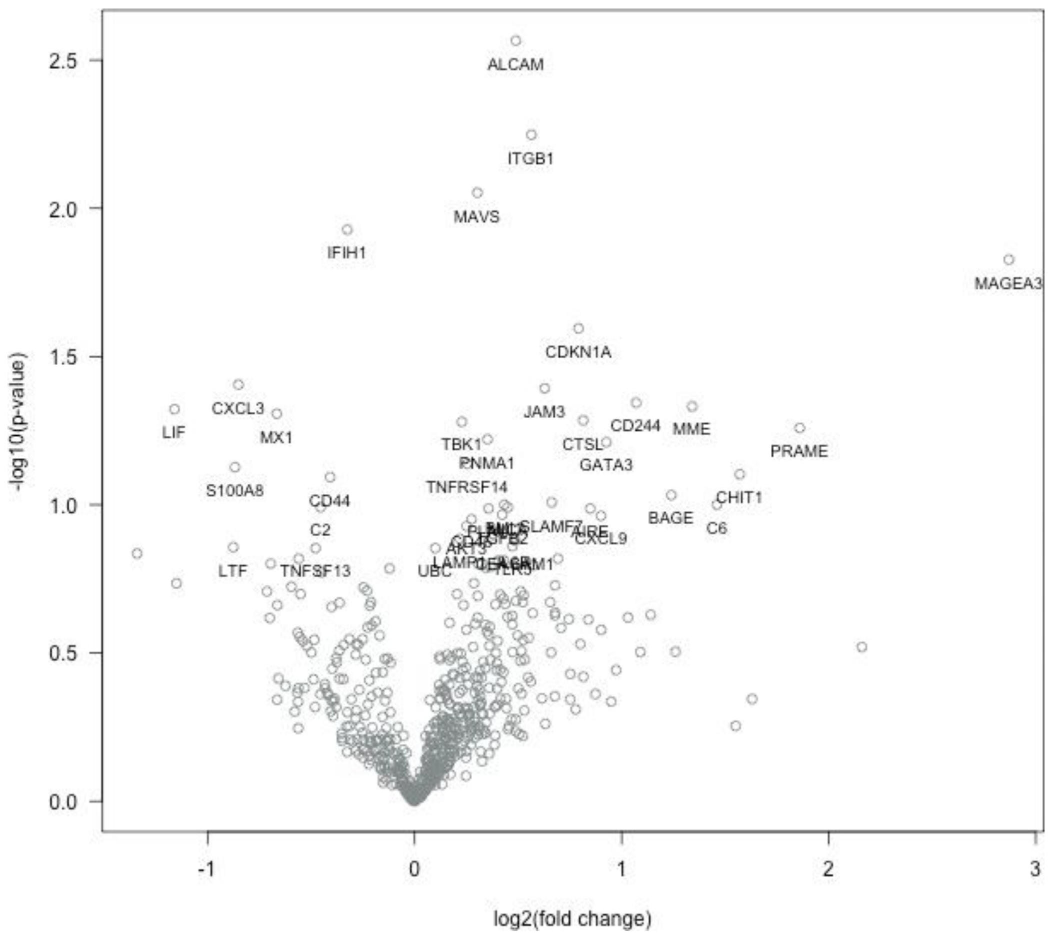

Supplementary Figure 2. A volcano plot of the differentially expressed immune genes in older versus younger patients by NanoString analysis.

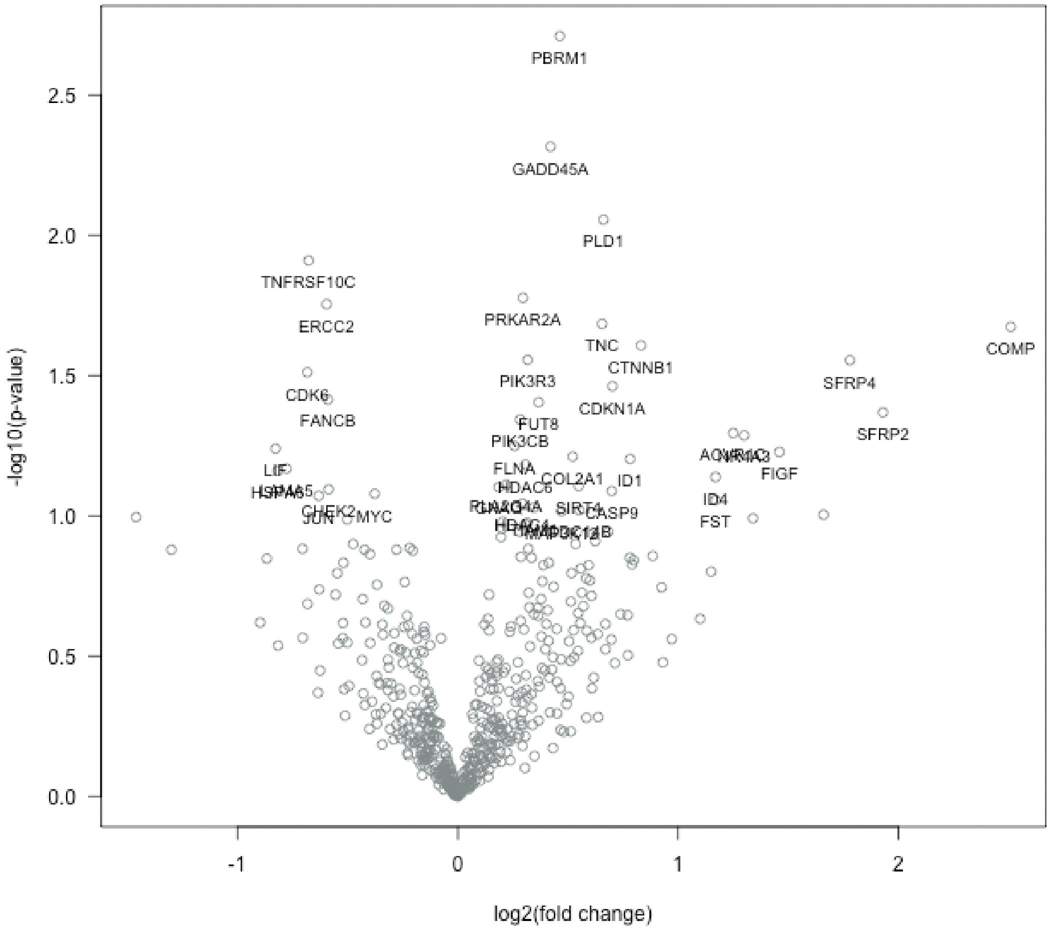

Supplementary Figure 3. A volcano plot of the differentially expressed immune pathway genes in older versus younger patients by NanoString analysis.

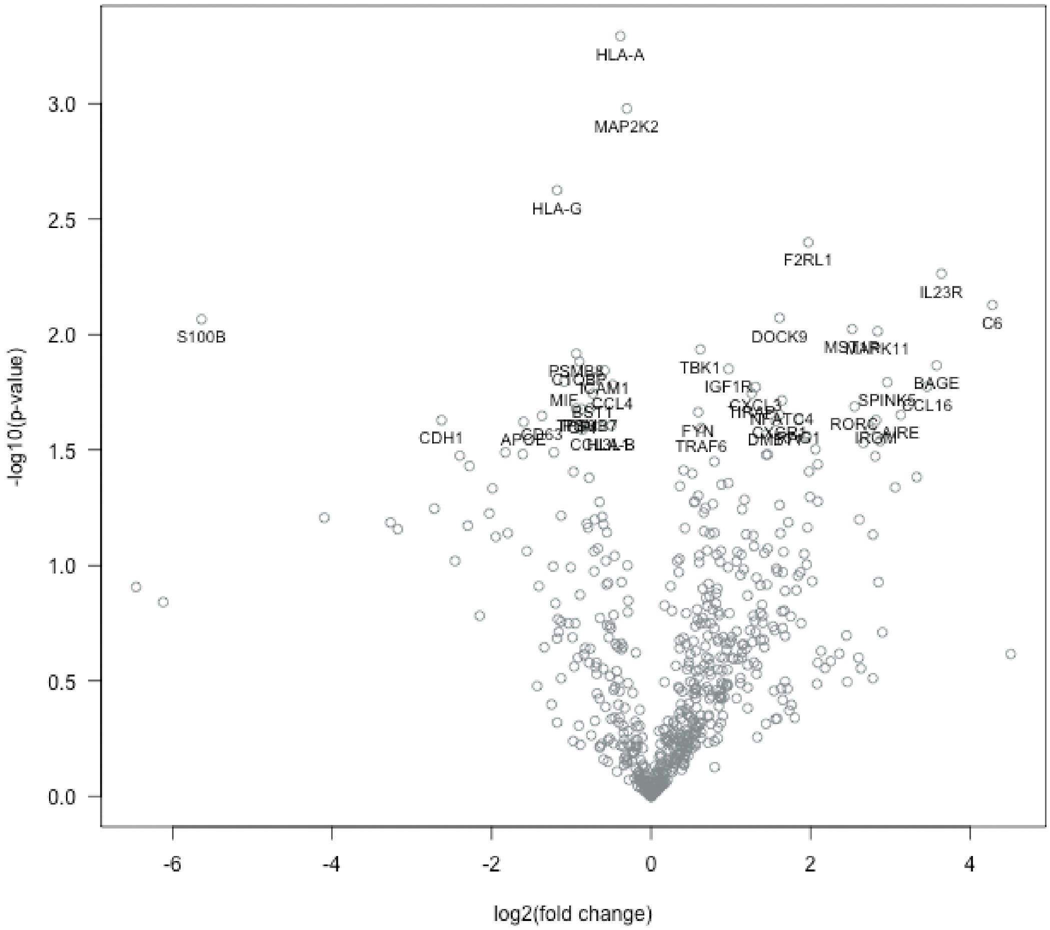

Supplementary Figure 4. A volcano plot of the immune genes that was differentially expressed in recurrence versus the no recurrence in the younger patient group.

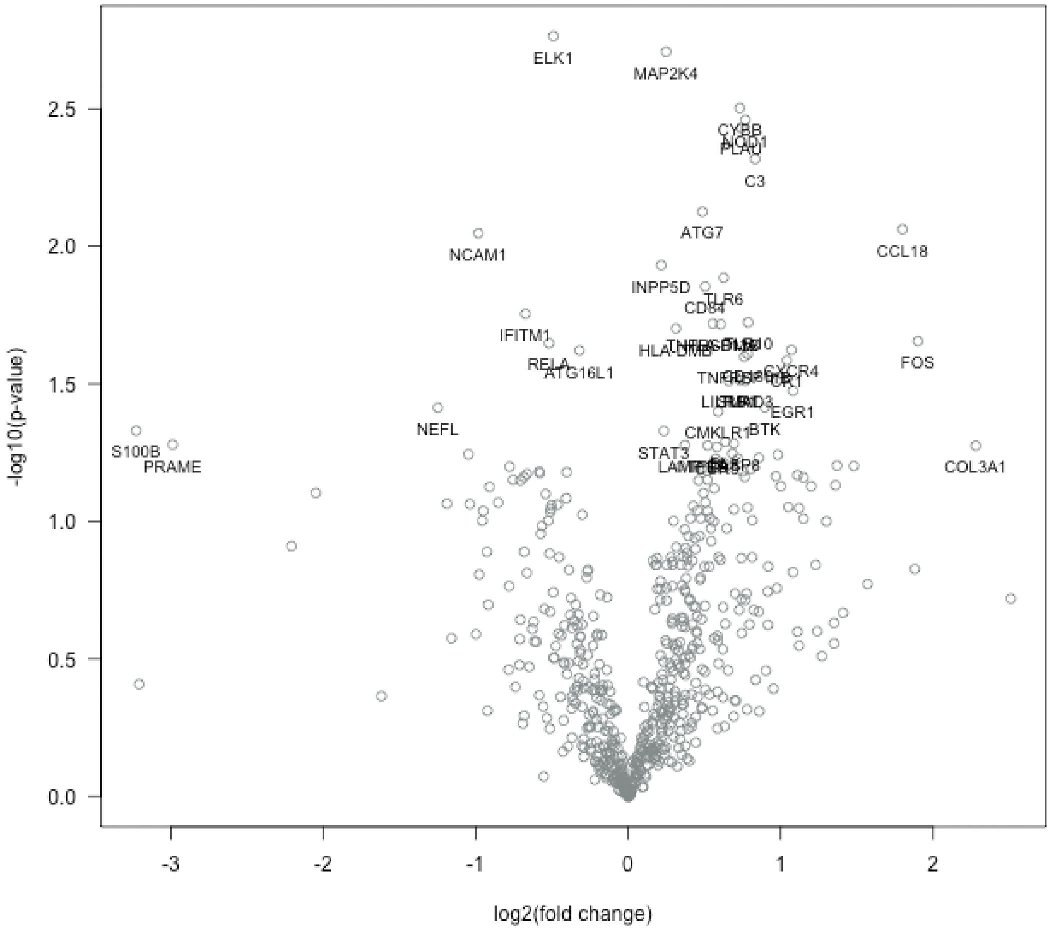

Supplementary Figure 5. A volcano plot of the immune genes that was differentially expressed in recurrence versus the no recurrence in the older patient group.

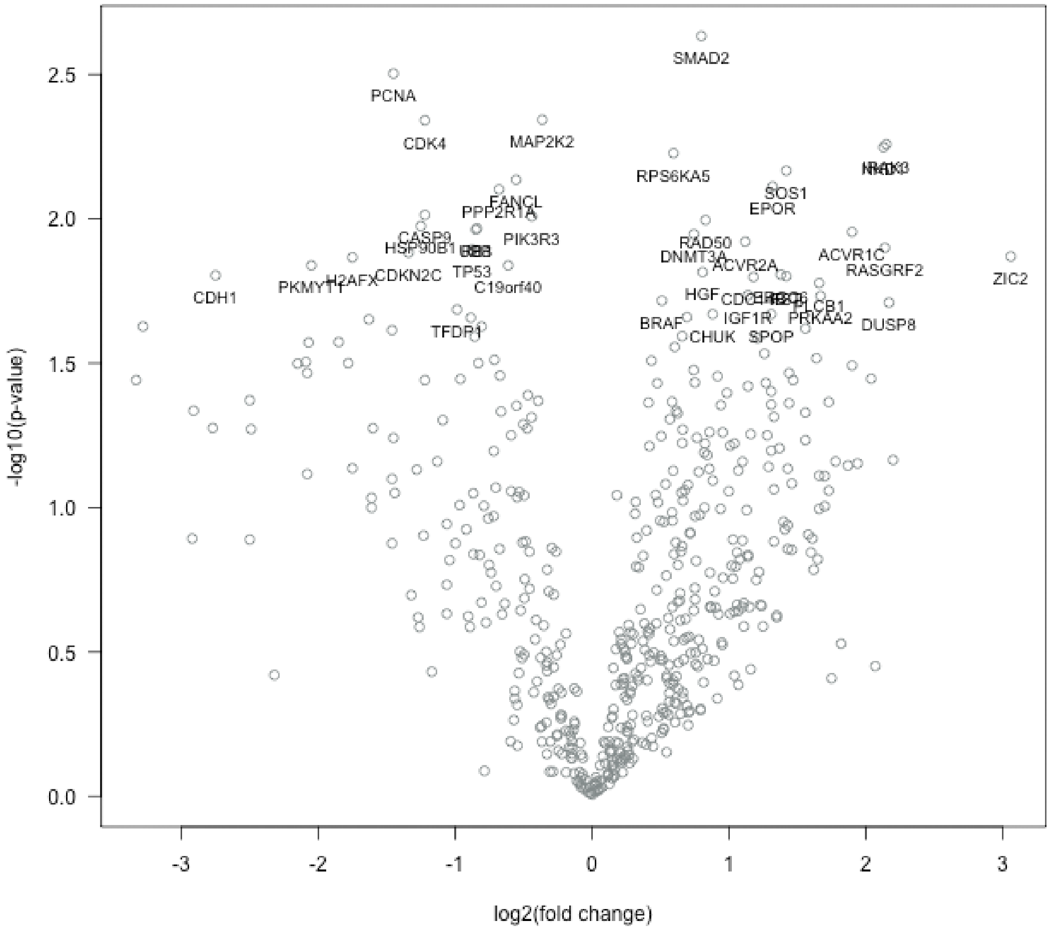

Supplementary Figure 6. A volcano plot of the immune pathway genes that was differentially expressed in recurrence versus the no recurrence in younger patients.

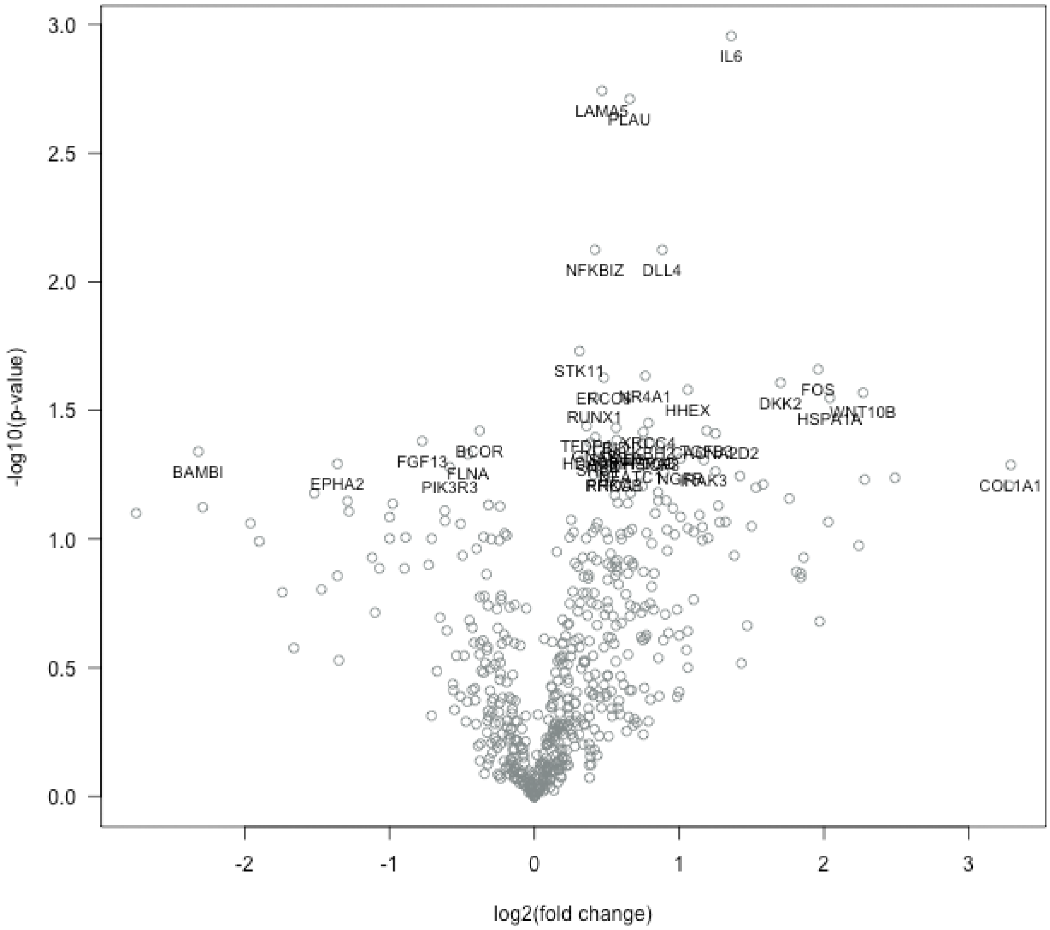

Supplementary Figure 7. A volcano plot of the immune pathway genes that was differentially expressed in recurrence versus the no recurrence in older patients.
