# Supplementary material for: Age-related transcriptome changes in melanoma patients with tumor-positive sentinel lymph nodes
Source: Aging (Albany NY). 2020 Dec 29;12(24):24914–39. doi: 10.18632/aging.202435 (PMC7803563; doi:10.18632/aging.202435)
Supplement: Supplementary Tables [file aging-12-202435-s002.pdf]

SUPPLEMENTARY TABLES

Supplementary Table 1. Top canonical pathways that showed differences in yr60+ versus yr60- patients in the first microarray dataset using T4 filter.

Pathway name	*p*- value	Overlap
PPAR Signaling	2.65E-04	4.0% (4/101)
Acute phase response signaling	2.20E-03	2.2% (4/178)
Melanocyte development and pigmentation signaling	3.13E-03	3.2% (3/95)
Coagulation system	5.18E-03	5.7% (2/35)
Cholecystokinin/Gastrin-mediated signaling	5.61E-03	2.6% (3/117)

Supplementary Table 2. Differentially expressed genes (DEGs) in the SLN in yr60+ versus yr60- patients in the first microarray dataset using T3 filter.

Gene symbol	Gene Name	*P* value	Fold change
**FOSB**	FBJ murine osteosarcoma viral oncogene homolog B	0.0208	1.5961
**FOS**	FBJ murine osteosarcoma viral oncogene homolog	0.0255	1.5558
**DUSP1**	dual specificity phosphatase 1	0.0453	1.5349
**NR4A2**	nuclear receptor subfamily 4, group A, member 2	0.0096	1.4719
**IDO1**	indoleamine 2,3-dioxygenase 1	0.0155	1.454
**CLEC4C**	C-type lectin domain family 4, member C	0.0486	1.4497
**LIX1**	limb and CNS expressed 1	0.0098	1.4118
**CD8A**	CD8a molecule	0.0003	1.4111
**BACH2**	BTB and CNC homology 1, basic leucine zipper transcription factor 2	0.003	1.3963
**NRCAM**	neuronal cell adhesion molecule	0.0008	1.3961
**NOG**	noggin	0.0027	1.3839
**KLRC4-KLRK1 /// KLRK1**	KLRC4-KLRK1 read through /// killer cell lectin-like receptor subfamily K, member 1	0.0011	1.3827
**MS4A6A**	membrane-spanning 4-domains, subfamily A, member 6A	0.0434	1.3709
**KLF4**	Kruppel-like factor 4 (gut)	0.0278	1.3662
**SATB1**	SATB homeobox 1	0.0322	1.3484
**LOC101928963**	uncharacterized LOC101928963	0.027	1.2614
**GRIK2**	glutamate receptor, ionotropic, kainate 2	0.0497	0.8748
**MUC15**	mucin 15, cell surface associated	0.0495	0.8681
**DLK1**	delta-like 1 homolog (Drosophila)	0.0339	0.8617
**RNF152**	ring finger protein 152	0.05	0.8483
**ITGBL1**	integrin beta like 1	0.03	0.8466
**ERGIC3**	ERGIC and golgi 3	0.0295	0.8456
**INHBA**	inhibin beta A	0.0461	0.8347
**PRUNE2**	prune homolog 2 (Drosophila)	0.0059	0.8323
**LINC00354**	long intergenic non-protein coding RNA 354	0.0292	0.8312
**MKX**	mohawk homeobox	0.0053	0.8308
**WWC1**	WW and C2 domain containing 1	0.0295	0.8228
**LOC105373225**	uncharacterized LOC105373225	0.029	0.8139
**SLC13A5**	solute carrier family 13 (sodium-dependent citrate transporter), member 5	0.018	0.7921
**GRB14**	growth factor receptor bound protein 14	0.0135	0.792
**ATP2B2**	ATPase, Ca++ transporting, plasma membrane 2	0.0295	0.791
**COL28A1**	collagen, type XXVIII, alpha 1	0.0092	0.7779
**LOC100507516**	uncharacterized LOC100507516	0.0223	0.7719
**MLANA**	melan-A	0.0486	0.7057

Supplementary Table 3. Differentially expressed immune genes in younger patients between the recur^{yes} and the recur^{no} group by NanoString analysis (p<0.05).

Gene symbol	Gene Name	*P* value	Fold change
**C6**	complement component 6	0.00745	4.28
**IL23R**	interleukin 23 receptor	0.00545	3.64
**BAGE**	B melanoma antigen	0.0136	3.58
**CCL16**	chemokine (C-C motif) ligand 16	0.0168	3.46
**SPINK5**	serine peptidase inhibitor, Kazal type 5	0.0161	2.96
**MAPK11**	mitogen-activated protein kinase 11	0.00968	2.84
**MST1R**	macrophage stimulating 1 receptor (c-met-related tyrosine kinase)	0.00947	2.52
**F2RL1**	coagulation factor II (thrombin) receptor-like 1	0.00399	1.97
**DOCK9**	dedicator of cytokinesis 9	0.00847	1.61
**IGF1R**	insulin-like growth factor 1 receptor	0.0141	0.971
**TBK1**	TANK-binding kinase 1	0.0116	0.616
**MAP2K2**	mitogen-activated protein kinase kinase 2	0.00105	-0.306
**HLA-A**	major histocompatibility complex, class I, A	0.00051	-0.386
**CCL4**	chemokine (C-C motif) ligand 4	0.0165	-0.485
**ICAM1**	intercellular adhesion molecule 1	0.0143	-0.587
**C1QBP**	complement component 1, q subcomponent binding protein	0.0131	-0.9
**PSMB8**	proteasome (prosome, macropain) subunit, beta type, 8 (large multifunctional peptidase 7)	0.0121	-0.939
**MIF**	macrophage migration inhibitory factor (glycosylation-inhibiting factor)	0.016	-1.09
**HLA-G**	major histocompatibility complex, class I, G	0.00237	-1.18
**S100B**	S100 calcium binding protein B	0.00859	-5.64

Supplementary Table 4. Differentially expressed immune genes in older patients between the recur^{yes} and the recur^{no} group by NanoString analysis (*p*<0.05).

Gene symbol	Gene Name	*P* value	Fold change
**FOS**	FBJ murine osteosarcoma viral oncogene homolog	0.0221	1.9
**CCL18**	chemokine (C-C motif) ligand 18 (pulmonary and activation-regulated)	0.00867	1.8
**CXCR4**	chemokine (C-X-C motif) receptor 4	0.0238	1.07
**C3**	complement component 3	0.00481	0.832
**TLR10**	toll-like receptor 10	0.0189	0.787
**NOD1**	nucleotide-binding oligomerization domain containing 1	0.00347	0.768
**PLAU**	plasminogen activator, urokinase	0.00371	0.741
**CYBB**	cytochrome b-245, beta polypeptide	0.00314	0.732
**TLR6**	toll-like receptor 6	0.013	0.626
**HLA-DMA**	major histocompatibility complex, class II, DM alpha	0.0192	0.606
**TNFRSF13B**	tumor necrosis factor receptor superfamily, member 13B	0.0191	0.555
**CD84**	CD84 molecule	0.014	0.504
**ATG7**	autophagy related 7	0.00748	0.486
**HLA-DMB**	major histocompatibility complex, class II, DM beta	0.0199	0.313
**MAP2K4**	mitogen-activated protein kinase kinase 4	0.00196	0.248
**INPP5D**	inositol polyphosphate-5-phosphatase, 145kDa	0.0117	0.216
**ELK1**	ELK1, member of ETS oncogene family	0.00172	-0.491
**RELA**	v-rel reticuloendotheliosis viral oncogene homolog A (avian)	0.0225	-0.52
**IFITM1**	interferon induced transmembrane protein 1	0.0176	-0.675
**NCAM1**	neural cell adhesion molecule 1	0.00896	-0.984

Supplementary Table 5. Differentially expressed immune pathway genes in younger patients between the recur^{yes} and the recur^{no} group by NanoString analysis (*p*<0.05).

Gene symbol	Gene Name	*P* value	Fold change
**IRAK3**	interleukin-1 receptor-associated kinase 3	0.00552	2.15
**NKD1**	naked cuticle homolog 1 (Drosophila)	0.00565	2.13
**ACVR1C**	activin A receptor, type IC	0.0111	1.9
**SOS1**	son of sevenless homolog 1 (Drosophila)	0.00681	1.42
**EPOR**	erythropoietin receptor	0.00773	1.32
**ACVR2A**	activin A receptor, type IIA	0.012	1.12
**RAD50**	RAD50 homolog (S. cerevisiae)	0.0101	0.83
**SMAD2**	SMAD family member 2	0.00233	0.799
**DNMT3A**	DNA (cytosine-5-)-methyltransferase 3 alpha	0.0113	0.745
**RPS6KA5**	ribosomal protein S6 kinase, 90kDa, polypeptide 5	0.00593	0.596
**MAP2K2**	mitogen-activated protein kinase kinase 2	0.00454	-0.363
**PIK3R3**	phosphoinositide-3-kinase, regulatory subunit 3 (gamma)	0.00981	-0.439
**FANCL**	Fanconi anemia, complementation group L	0.00732	-0.554
**PPP2R1A**	protein phosphatase 2, regulatory subunit A, alpha	0.00791	-0.679
**RB1**	retinoblastoma 1	0.0108	-0.84
**UBB**	ubiquitin B	0.0109	-0.849
**CDK4**	cyclin-dependent kinase 4	0.00456	-1.22
**CASP9**	caspase 9, apoptosis-related cysteine peptidase	0.00969	-1.22
**HSP90B1**	heat shock protein 90kDa beta (Grp94), member 1	0.0106	-1.25
**PCNA**	proliferating cell nuclear antigen	0.00314	-1.45

Supplementary Table 6. Differentially expressed immune pathway genes in older patients between the recur^{yes} and the recur^{no} group by NanoString analysis (*p*<0.05).

Gene symbol	Gene Name	*P* value	Fold change
**WNT10B**	wingless-type MMTV integration site family, member 10B	0.027	2.27
**HSPA1A**	heat shock 70kDa protein 1A	0.0283	2.04
**FOS**	FBJ murine osteosarcoma viral oncogene homolog	0.0219	1.96
**DKK2**	dickkopf WNT signaling pathway inhibitor 2	0.0247	1.7
**IL6**	interleukin 6 (interferon, beta 2)	0.00111	1.36
**TGFB3**	transforming growth factor, beta 3	0.0379	1.19
**HHEX**	hematopoietically expressed homeobox	0.0263	1.06
**DLL4**	delta-like 4 (Drosophila)	0.00752	0.883
**XRCC4**	X-ray repair complementing defective repair in Chinese hamster cells 4	0.0354	0.787
**NR4A1**	nuclear receptor subfamily 4, group A, member 1	0.0232	0.766
**ALKBH2**	alkB, alkylation repair homolog 2 (E. coli)	0.0384	0.752
**PLAU**	plasminogen activator, urokinase	0.00195	0.659
**BID**	BH3 interacting domain death agonist	0.0369	0.564
**ERCC6**	excision repair cross-complementing rodent repair deficiency, complementation group 6	0.0236	0.479
**LAMA5**	laminin, alpha 5	0.00181	0.467
**NFKBIZ**	nuclear factor of kappa light polypeptide gene enhancer in B-cells inhibitor, zeta	0.00751	0.418
**RUNX1**	runt-related transcription factor 1	0.0281	0.417
**TFDP1**	transcription factor Dp-1	0.0364	0.36
**STK11**	serine/threonine kinase 11	0.0186	0.312
**BCOR**	BCL6 corepressor	0.0379	-0.377

Supplementary Table 7. Patients' data on age and outcome in all three datasets.

Outcome	1st microarray dataset		2nd NanoString dataset		3rd dataset qRT-PCR	
	< 60	≥ 60	< 60	≥ 60	< 60	≥ 60
No recurrence ($recur^{no}$)	51	7	3	3	9	13
Recurrence ($recur^{yes}$)	28	11	3	3	9	5
